# Hospitals for Children

**Published:** 1887-08-06

**Authors:** 


### Hospitals for Children.

In all London there is no more touching and suggestive sight than that presented by the wards of its children's hospitals. Only, to fully understand them, one ought by right to see not only the wards in which the children lie, but the homes and the lives from which many of them have come. Here they are gathered in beautiful halls—light, airy, cheerful, and scrupulously clean. These establishments—of which there are ten or a dozen now in London—are not all equally showy in appearance, but stained glass windows, artistically painted walls, toys, pictures and picture-books, scrap-albums, flowers and musical-boxes, are among the details that go to make up the *tout ensemble* that meets the eye of the visitor who steps into many of the London hospitals devoted to the ailments and accidents of children. There the little sufferers lie, each in its cot with its snowy coverlet, each child—if able to sit up—in a bright crimson or scarlet flannel jersey, and many of them looking as rosy and happy as though nothing ailed them, and as though they had not a wish in the world but to be allowed always to live in what to many of the little mortals must be a veritable fairyland. A children's hospital ward almost always affords a great surprise to those who visit it for the first time. It is not the sickness and suffering that first obtrudes itself on the attention; it is the brightness and pleasantness of the place, the pretty appearance of the children, and the general air of order, sweetness, and comfort.

Many of the inmates, too, are really most diverting little things. I did not see the child, but I had the other day a photograph of a little girl, of whom an anonymous writer who had been to the hospital a little before my visit, says that, whenever she entered the ward, she was usually invited to a game of doctors. The visitor was to be the nurse, and Nellie the doctor, and an old black doll the patient. "How is she today?" was the little doctor's first question. "Much the same, I think." "Let me look at her leg." A careful examination of a diminutive stuffed limb now followed, and then came a solemn order for lint and a tight-bandage. Dolly, however, always resented the treatment prescribed, and Nellie—who had gone through it all herself, and understood exactly what dolly must feel—was profuse with her kisses and caresses and soothing words. After a while the patient was pronounced well enough to go to the "ton'lescent ward"—the convalescent ward, that is. I stood the other day by the bedside of quite an original little character, a child apparently of about three to four years old, a wizened, wasted little figure, suffering from a complication of diseases, and wheezing, and panting for breath like a badly asthmatical old man, yet evidently as full of fun and humour as a Mark Tapley or a Joe Miller. Bronchitis, inflammation of the lungs, and rickets, were among his maladies. During the night hours I was told he would be quite exhausted by a desperate struggle for breath, but in the morning would be found bright and cheery as though nothing had been the matter. He put out a wasted little hand for me to shake, and tried to tell me his name, but his breath was not equal to it, and he fell back on his pillow and gasped, and then seemingly misunderstanding something said to him by the sister who was attending him, his ancient little face broke into a humorous grin, and

> "He put his thumb unto his nose,
> And spread his fingers out."

It was dreadfully ill-mannered, of course, and the sister was shocked; but it was so unexpected, and there was something so irresistibly comic in the exuberance of fun in this little wasted sufferer, that I could not but laugh heartily. This little one's appearance and happy flow of spirits reminded me of another tiny patient I had seen on another occasion. Poor little chap, he was sadly afflicted, and face and form bore distressing evidence of it; but it was very droll to see him roll up his shirt-sleeves, bare a lilliputian pair of arms, and make a furious show of fight with anybody who would entertain him with a round or two in a friendly way. The nurses and sisters seem fond of encouraging little ecentricities of this sort, though it often happens that they have to laugh in their sleeves at some of the infantile freaks of humour. I saw the other day an intelligent-looking child, a girl, I suppose about six or seven years old. *Sub rosâ*, I was told that if anything were done to hurt her she would cry out: "Oh! don't. If you do that I shall be obliged to say d——n!" Very distressing indeed it is, I am told, sometimes to hear the language the merest babies will give utterance to in delirium. On the other hand, I had pointed out to me in one of the hospitals a child in a hopeless condition with tumour on the brain, and who lay in her tiny cot looking the very picture of happiness, and through almost all her waking hours quietly singing to herself a well-known ditty, "Jogging along."

Numbers of these children in all the hospitals come from homes of the very worst character, and pathetic proofs of the want and misery, the degraded brutality, the vice and ignorance prevailing in them are often betrayed by little incidents of vivid significance. I shall not soon forget the sad, dejected, unchild-like face which one Christmas Eve peered out at me from a cot in the Evelina Hospital. His mother had been to see him that day, and she had dropped a tear or two as she bent over him. "What's the matter, mother?" asked the child anxiously. "Has father been beating you again?" The evidences of ignorance and neglect on the part of parents are often very painful. "Look at that child," said the medical superintendent of the East London the other day: "we sent her out a fortnight ago in fair health and condition; now look at her! She has just been brought back again." Certainly, the most uncanny-looking baby I ever saw. It was like a monkey, without the monkey's hair. Its mouth was prominent; its tiny cheeks puckered in lines, and its forehead wrinkled like an old man's. Poor bairn! it was doing its best to fill out the puckers and the wrinkles with the aid of a feeding-bottle, but there was the feverish, worried look upon the little face, as if conscious that it was all behind with its work, and could never fetch up arrears. "Really," said Sir James Paget at a meeting in connection with one of these institutions, "the imprudent marriages, the intemperance, the reckless poverty, the dirt and squalor of parents, do not tell their bitterest tale in their own unhappy lives at home. They tell it much more severely in their offspring." True; alas! too true. These diseased hips and rickets and crooked spines, these "wasting" children—"wasters," as they are called in hospital parlance—these skin diseases, and a large proportion of the accidents, too, are for the most part due to carelessness and ignorance, to vice and poverty, and neglect. Thousands of the homes from which these cases come are a burning disgrace to our civilisation, and upon these helpless little ones the brunt of the mischief falls.

One cannot but feel profoundly thankful for these institutions, while "homes" are so often what they are. But if there is one feature of them more than another calculated to evoke that thankfulness it is the operating room. What those dreadful chambers must have been in those dark, sad days of hospital mismanagement to which we have alluded in previous articles, it is sickening indeed to think of. I remember well, the first time I ever stepped into the operating theatre of a children's hospital, what a thrill of horror ran through me, and with what a profound sense of relief came the next moment the thought of chloroform. Since then I had a memorable afternoon in the operating theatre of one of the large general hospitals, and among other operations I witnessed was a severe one on an infant. I have often reverted in my mind to that afternoon's experience with profound satisfaction. To most of us who are not inured to the kind of thing, there is something very gruesome in the idea of a surgical "operation," and few persons have ever anticipated such a spectacle with greater trepidation than I did. Had I been aware that a delicate child was to be one of the subjects, I do not think I could have mustered fortitude; but the sliding doors were pushed open, and, all unexpected by me, the little thing was borne in upon a palliasse, apparently in an insensible condition. It is, I believe, the practice in such cases to administer an anæsthetic in the ward, and so to reduce the patient to a semi-unconscious state before it is borne away to the operating room. That appeared to have been done here, and when the little thing had been conveniently adjusted on the table it was further narcotised by chloroform. Then there were a few brief observations addressed to the assembled students, and without anything calculated greatly to distress the most nervous, the operation was quietly and deftly performed, the wound dressed, and the unconscious little subject borne back to bed, still under the influence of the anæsthetic, and likely to slumber through the worst of the period attending so severe an ordeal. That afternoon's experience has for ever robbed the children's hospital of its greatest terror to my mind; and though it must always be unutterably sad to move from bed to bed amid these little martyrs, it is a sadness by no means unmingled with pleasure as one mentally contrasts these conditions with what it would have been in days not so very long ago. How strange it is that with all the loving sympathy these institutions have for their tiny inmates, they are almost all of them hampered and hindered in their beneficent work for want of funds, and that every day in the year little sufferers are turned from their doors, many of them sent back to homes of poverty and wretchedness, because the kind-hearted, comfortably-off parents of England cannot by any practicable means be aroused to an adequate sense of their need of help!

